# Good Food, Good Mood: Perspectives on the Relationship Between Nutrition and Mental Health With Division I Collegiate Athletic Programs

**DOI:** 10.3389/fspor.2021.692601

**Published:** 2021-07-21

**Authors:** Emma M. McCabe, Caroline J. Ketcham, Eric E. Hall

**Affiliations:** ^1^Department of Exercise Science, Elon University, Elon, NC, United States; ^2^Elon BrainCARE Research Institute, Elon University, Elon, NC, United States

**Keywords:** nutrition, mental health, athletic programs, athletes, sport psychology

## Abstract

Research has shown a strong relationship between nutrition and mental health. Packed schedules and little rest time may make student-athletes more susceptible to mental health issues than the general population, but few athletes are fully aware of the effects that nutrition can have on their mental health. While collegiate athletic programs are beginning to recognize the individual contributions of nutrition and mental health to performance and are hiring sport dietitians and psychologists for their athletes, it is unclear whether these topics are ever discussed within the same context. The goal of this study was to understand the perspectives of different athletic personnel on the relationship between nutrition and mental health. 17 athletic personnel (11 Female, 6 Male) from 6 NCAA Division I universities were recruited for a 30–45-min semi-structured WebEx interview. Participants included athletic trainers, coaches, dietitians, sport psychologists, strength and conditioning coaches, and sports medicine physicians. Participants were asked questions about their educational backgrounds, resources, and perspectives on the integration of nutrition and mental health in their programs. Transcribed responses were sorted into four themes: (1) Resources, (2) Education, (3) Department Integration or Collaboration, and (4) Student and Coach Engagement. All participants reported a need for greater monetary resources and staffing. Around 59% of the participants felt they had little more than general or personal interest-level knowledge on topics pertaining to nutrition or mental health, with the exception of sports dietitians or psychologists. Each school varied in the degree to which their athletic staff regularly communicated about their work and athlete health statuses. Athletes were reportedly more or less likely to utilize the resources provided depending on coach attitudes toward nutrition or mental health. Regardless of size, reputation and annual spending, each university was reported to be in the early stages of integrating nutrition and mental health programs into their existing athletic departments. Implications of this work may be to help schools plan for ways to reallocate funding for nutrition or mental health programming.

## Introduction

In the United States (U.S.), participating in a college varsity sports program is often considered the pinnacle of a student's athletic career (Popp et al., 2009). U.S. college athletes seem to base their university experience on their athletic successes (Popp et al., 2009). Therefore, student-athletes are supported by a collaborative network of athletes, coaches and trainers, parents and staff that work toward the main goal of optimizing athletic performance. Evidence shows that a long-term, balanced diet is key to optimal brain function and mental health (Rucklidge and Kaplan, 2016; Owen and Corfe, 2017; Firth et al., 2020). However, not many athletes in the U.S. are fully aware of the effect that diet can have on their brain (Parks et al., 2016; Abbey et al., 2017). In fact, fewer athletes have proper training in general nutrition and its impact on performance (Parks et al., 2016; Abbey et al., 2017). Many athletic programs have begun to incorporate both nutrition and mental health services and education into their athletic experiences, with a focus on health for the sake of improving performance outcomes (Parks et al., 2016; Rudick and Dannels, 2018). However, changes in public perception of the health needs of student-athletes have pushed colleges to further build their athletic departments, and take on the role of caring for athletes' whole health (NCAA, 2020).

There is evidence that nutrition plays a role in developing and altering brain function (Sharma et al., 2012; Rucklidge and Kaplan, 2016; Wong et al., 2016; Owen and Corfe, 2017; Kapczinski et al., 2019; Marx et al., 2019; Firth et al., 2020). In particular, connections have been drawn between a high-fat diet (HFD) and anxiety or depressive disorders (Sharma et al., 2012; Wong et al., 2016; Kapczinski et al., 2019; Marx et al., 2019). The common “Western diet,” consisting of high calories and processed foods with high saturated fat, is a HFD connected with high levels of inflammation and declines in cognitive function (Wong et al., 2016; Noble et al., 2017; Kapczinski et al., 2019). A pivotal human trial assessing the effect of diet on mental health revealed that, after accounting for lifestyle choices, participants with traditionally “whole food” diets including fish, fruits and vegetables reported lowest levels of depression after 5 years, while those consuming a more westernized HFD reported the greatest depression levels (Marx et al., 2019).

Transitioning college students experience new changes and stressors, including access to new food choices. Research indicates that college diets are often low in energy, high in fat, and deficient in several micronutrients (Driskell et al., 2008; Roy et al., 2017; Sunbul et al., 2019), reflective of an HFD. This may be due to the fact that many U.S. colleges have buffet-style dining halls, giving students limited hours for eating and overwhelming options to choose from, many of which are not the most nutritional (Driskell et al., 2008; Wengreen and Moncur, 2009; Roy et al., 2017). Given that collegiate student-athletes fall under this population, it is essential that athletes be educated on general nutrition and benefits that a well-balanced diet can have on their physical, as well as mental, health and performance. Nutrition and mental health have recently taken center stage in the world of college athletics (Parks et al., 2016; Rudick and Dannels, 2018). The NCAA has declared mental health support one of its top priorities for athletics (Henry, 2020), as the rising number of mental health diagnoses and suicides on college campuses has been labeled as a major public health concern (Liu et al., 2019). For instance, over 30% of 950 NCAA Division I student-athletes experience symptoms of depression (Brown, 2014; Cox et al., 2017), and over 50% have experienced “overwhelming” anxiety in the last year (Brown, 2014). The use of counseling services in colleges across the U.S. has increased significantly, with over half of students who seek help leaving with a severe psychological diagnosis (Rudick and Dannels, 2018). It still remains, however, that less than half of student-athletes who feel concerned with their mental health during their college career will seek out help from their institution. Many schools have also begun screening athletes for mental health disorders (Kroshus, 2016). A study that examined the number of schools with existing protocols found that under half of all NCAA schools reported having a screening protocol as of 2016, in which sports medicine staff administered a written or verbal screening instrument for common mental health issues like eating disorders, depression or anxiety (Kroshus, 2016). A separate survey of athletic trainers from 336 NCAA DI schools between 2015 and 2016 found similar results, with less than half reporting use of screening tools (Sudano and Miles, 2016).

Advancements are being made in the world of nutrition as well. The number of U.S. collegiate programs with a registered sports dietitian has nearly quadrupled in the last decade (Parks et al., 2016), although studies have shown that athletes continue to rely primarily on athletic trainers, coaches and online searches for nutritional knowledge (Abbey et al., 2017). Studies with athletic trainers and coaches have revealed that their nutritional knowledge is still lacking (Corley et al., 1990; Torres-McGehee et al., 2012; Trakman et al., 2016). Since athletes are likely to interact with coaches and athletic trainers on a daily basis, the training of these staff members in general nutrition and mental health topics is essential (Torres-McGehee et al., 2012). Several studies on nutrition knowledge within U.S. college athletic programs have focused on the need for increased nutritional training and new perspectives on nutrition in the field of sport (Corley et al., 1990; Abood et al., 2004; Torres-McGehee et al., 2012; Andrews et al., 2016; Trakman et al., 2016). For instance, one 2016 study among 123 Division I schools found that the majority of students had inadequate (less than 60%) nutrition knowledge, with no differences by sex, class level, team or prior nutrition education (Andrews et al., 2016). Athletic trainers and strength and conditioning coaches have been found to have the most adequate nutrition knowledge aside from sports dietitians, but collaboration between sports medicine and sports nutrition staff is recommended (Torres-McGehee et al., 2012). Reviews of studies examining coach and athlete nutrition knowledge found that knowledge was especially lacking in areas involving protein consumption for different diets, the roles of protein in muscle-building and performance, sources of fat, energy density and vitamin or mineral supplementation (Trakman et al., 2016). A recurring theme among most of these studies was that prior education was correlated with improved knowledge scores and dietary behaviors (Valliant et al., 2012; Trakman et al., 2016), suggesting that schools with access to nutrition staff or counseling may be able to offer better care for their athletes. Interventions have been attempted to improve general nutrition knowledge among athletes (Abood et al., 2004), but integration of nutrition staff and programming will likely result in greater overall exposure to nutrition topics and make a more lasting impact on athletes. Since nutrition is important for maintaining both physical and mental health (Andrews et al., 2016; Rucklidge and Kaplan, 2016; Owen and Corfe, 2017; Firth et al., 2020), the addition of nutrition education to DI athletic programs, staff and students is strongly recommended. Many U.S. sports programs have begun to recognize the individual and cumulative effects that diet and mental health can have on athlete performance, but it is unclear whether nutrition and mental health are ever thought of and discussed within the same context. The increasing emphasis of having both nutrition and mental health resources incorporated into collegiate athletic programs presents a unique opportunity to improve upon the knowledge of both athletic personnel and athletes on the roles of nutrition and mental health, and to create an integrative athletic team that focuses on the whole needs of each athlete. It is therefore important that a baseline profile of NCAA schools exists on the level of integration that exists between nutrition and mental health in their athletic programs. From there, NCAA schools can be made aware of the status of other schools' programs, as well as the major changes that need to be made in order to better serve their student-athletes. Since Division I programs are currently the most advanced in terms of the ratio of staff to athletes (Baugh et al., 2020), these schools were selected for this study.

Each school may have different populations, access to resources, and perspectives on the roles and interaction of nutrition and mental health. Therefore, it is possible to make comparisons between the perspectives of these programs on the role of nutrition and its effect on the mental health of these college athletes. The purpose of this study was to examine the perspectives of athletic personnel involved in NCAA Division I programs, on knowledge and education of the link between diet and mental health.

## Materials and Methods

### Participants

Seventeen athletic and medical personnel (9F, 6M, 2 N/A) across 6 NCAA Division I programs participated in the study. Table 1 lists the demographic characteristics of each school. Four athletic trainers, 2 sports dietitians, 5 sports psychologists, 1 head coach, 1 assistant coach, 3 strength and conditioning coaches and 1 sports medicine physician participated in the study. All but one participant were non-Hispanic white. Two participants did not complete the demographics survey and therefore did not report their gender, race, or ethnicity. The study's procedures were reviewed and approved by the Institutional Review Board (IRB) of Elon University.

**Table 1:** Demographic characteristics of the schools where participants worked, including: number of each type of athletic staff interviewed, public or private status, number of undergraduate students, number of athletes, number of athletic teams and total reported spending per year on athletics.

**Institution**	**Participants**	**Other information**
School A	1 athletic trainer	Public
	1 sports dietitian	21,000 students
	1 sports psychologist	>650 athletes
	1 coach	21 athletic teams
	2F, 1M, 1 N/A	$44.32 million spent per year on athletics
School B	2 athletic trainers	Private
	1 behavioral psychologist	>6,000 undergraduates (>15,000 Students Total)
	1 sports dietitian	>600 athletes
	3 F, 1 M	23 athletic teams
		>$78 million spent per year on athletics
School C	1 athletic trainer	Private
	1 coach	>6,000 undergraduates
	1 strength and conditioning coach	400 athletes
	1 sports medicine physician	16 athletic teams
	2F, 1M, 1 N/A	$18–19 million spent per year on athletics
School D	2 sports psychologists	Public
	1 strength and conditioning coach	>19,000 undergraduates
	1F, 2M	>500 athletes
		16 Athletic teams
		$34–35 million spent per year on athletics
School E	1 sports psychologist (F)	Public
		16,000 undergraduates
		>250 athletes
		13 athletic teams
		$10–11 million spent per year on athletics
School F	1 strength and conditioning coach (M)	Public
		>15,000 undergraduates (over 24,000 students total)
		450 athletes
		18 athletic teams
		$18 million spent per year on athletics

### Methodology

Fifty-nine prospective participants were identified through the staff directory of 11 NCAA Division I schools in the U.S. during spring 2020. Only head coaches, assistant coaches, strength and conditioning coaches, athletic trainers, sports dietitians, sports psychologists, sports medicine physicians, and athletic directors were selected for recruitment into the study (Table 1). Each prospective participant received an interest email which introduced the purpose of the research project. Seventeen participants showed interest in participating in the study.

Interested participants were then sent an email containing the Consent Form, which they were asked to read, sign and email back to the researcher. All signed consent forms were stored in a password-guarded Google Drive account. Meeting times were also decided via email, after which each participant received a unique WebEx (Milpitas, CA) invitation with a meeting link, number and password. Each participant completed a one-on-one, 30–45-minute, semi-structured interview that asked questions on their educational background, resources and perspectives on the integration of nutrition and mental health in their programs. Ten interview questions were previously created and agreed upon by the authors, with the goals of describing: (1) resources available to each institution for their nutrition and mental health programs; (2) participants' educational history; (3) participants' current level of nutrition and mental health knowledge; (4) participants' confidence levels when educating athletes on nutrition or mental health topics; (5) the organization of each institutions' nutrition and mental health programs; and (6) collaboration among athletic staff toward student-athlete health. Table 2 lists the questions asked to each participant. Questions were formatted and presented in a way to limit all implicit and explicit bias in participant responses. All interviews were individually conducted by a single moderator, but all authors met regularly to review the methods, recordings and transcripts for any biases and emerging themes. Following the interview, participants were asked if they had knowledge of other programs or personnel who could be contacted for an interview for this project and received a $20 electronic gift card for their time.

**Table 2:** Interview questions asked to each participant.

**Number**	**Main and supplemental questions**
1	What is your specialty/profession within your sports organization?
	a. Tell me about your typical workday, and how often you interact with athletes on a daily basis.
2	Tell me about your history with this athletic institution.
	a. How many years have you been in your current position?
3	Tell me about your educational background.
	a. Do you have any undergraduate and/or postgraduate degrees, as well as participation in any continuing education tracks?
4	Have you received any education or training on nutrition, as it pertains to athletics or otherwise?
	a. Have you taken part in any continuing education tracks?
	b. If so, do you use this knowledge in your current position?
5	Tell me about your definition of mental health. Have you received any education or training on mental health, as it pertains to athletics or otherwise?
	a. Have you taken part in any continuing education tracks?
	b. If so, do you use this knowledge in your current position?
6	Have you received any education or training on the relationship between nutrition and mental health?
	a. Have you taken part in any continuing education tracks?
	b. If so, do you use this knowledge in your current position?
7	Tell me about how nutrition and mental health are handled in your organization.
	a. Tell me about any resources that are available for you and/or athletes in relation to this information?
8	Do you feel comfortable/confident delivering nutrition and/or mental health information?
	a. Have you been given resources to assist in the provision of this knowledge?
9	Are you satisfied with the way nutrition and/or mental health education are handled in your profession and/or organization?
	a. What would be helpful to improve this relationship?
10	Are there any models of integration for nutrition and/or mental health information that you wish to adopt?
	a. Are there any organizations that you feel are incorporating nutrition and/or mental health well for their athletes?

*All questions had at least one supplemental or clarifying question asked, which are also included above*.

The first two of the 17 interviews were recorded using a personal laptop computer (2013 MacBook Air, Apple, Cupertino, CA) and were transcribed manually. The additional 15 interviews were recorded and stored on WebEx (Milpitas, CA), and were then sent to Rev.com (Austin, TX) for transcription. All transcripts were stored in a password-guarded Google Drive account, which could be accessed only by the authors. Recordings were kept in a password-guarded WebEx account (or on a personal, password-guarded laptop computer) so that they could be referenced to ensure transcript accuracy, or to check for any bias in the presentation of the interview questions. Qualitative analysis was completed by identifying the common themes that occurred during these conversations and assigning specific quotes to those themes. A list of potential themes was first identified by the first author (EMM) and discussed with the other authors following the interviews. All authors agreed upon these themes. Once no new themes began to emerge from the interviews, it was deemed that there was a saturation of responses and no new interviews took place. After the themes were identified, the first author went through the transcriptions and pulled quotes that best represented those themes. The major themes discussed during this research project were: Resources, Education, Department Integration or Collaboration, and Student and Coach Engagement. Each theme could be broken into several subcategories or “sub-themes” to which a minimum of three quotes applied. No specialized coding software was used for qualitative analysis. All the themes, sub-themes and quotes were recorded in a single, password-protected Google document. All participant names and school names were de-identified during analysis. If similar information was contained in quotes by multiple professionals for a school, we opted to use one representative quote. Results include quotes representative of multiple perspectives and content within each theme as they occurred, but an effort was made not to be duplicative.

## Results

### Resources

All staff asked for more resources, including both staffing and monetary contribution. For schools A, C and D, only one nutritionist and one sports psychologist existed for anywhere from 400 to 700 athletes.

“*There's one of me here for six to 700 athletes.”*—Sports Dietitian (A)

“*There's only one [sports psychologist]…there's 400-some athletes…The only other resource is the counseling center…then you're fighting over 20,000 other students for time with a counselor…Nutrition-wise, there's only two RD's.”*—Strength & Conditioning Coach (D)

“*I know that from talking with the behavioral health department that we need more staff…they're working beyond 40 hours per week and one person is part-time.”*—Athletic Trainer (B)

A few schools utilized master's or PhD students in place of full-time staffing.

“*We have master's students in our applied sports psychology program that we're doing work with some of the…teams”*—Sports Psychologist (E)

Three of the schools expressed difficulty retaining part and full-time staff due to coronavirus concerns and monetary limits at the university level.

“*We've been lucky enough to usually have a nutritionist to send people to, I don't know if that's the case anymore after the COVID, we might be downsizing.”*—Athletic Trainer (C)

“*I had a part-time dietitian this past year that was supposed to move into a full-time role, but there's a hiring freeze right now.”*—Sports Dietitian (A)

Some coaches and athletic trainers expressed a difference in access to resources between the typical “revenue” sports and less popular sports. Furthermore, some participants expressed that the same resources offered to student-athletes were limited to staff.

“*I think the major [revenue] sports have a completely different interaction with nutrition…that's one area where you see the haves and have not come out stronger.”*—Athletic Trainer (B)

“*Staff-wise…I don't think we have access to enough…If your own house isn't in order, that's hard to be a good coach or a good support staff”*—Strength & Conditioning Coach (D)

No school gave its club or intramural athletes the same access to sports nutrition and sports psychology services that are currently given to varsity athletes.

“*Club/intramural athletes unfortunately don't have the same resources…Our cheer team is sometimes an exception.”*—Sports Dietitian (B)

### Education

Although each individual felt confident in their own field of work, almost none of the participants felt they had more than general or personal interest-level knowledge on topics pertaining to nutrition or mental health, with the exception of registered dietitians or sports psychologists.

“*I know enough [about nutrition] to be dangerous.”*—Sports Psychologist (A)

“*There is a lot of interest and importance [in nutrition]. It's just the way I explain it isn't like a dietician. More general knowledge, and when I need to refer out, I have great resources.”*—Strength & Conditioning Coach (F)

For half of the schools it was reported that the university often provided opportunities for learning and training, but participants from the remaining schools reported having to look elsewhere for continuing education options.

“*The university itself is always sending out…educational and training options and opportunities for us to expand our knowledge in different areas… Especially during this time.”*—Sports Dietitian (A)

“*I wish they did [require additional training for coaches]. There's been some coaches meetings…it was just more of ‘This is what I would do, but this is what I would do,' but we never got a full answer, like, ‘Okay, but this is what you should do.'”*—Coach (A)

“*To be honest, there's been no talks about nutrition stuff. In regards to the mental health aspect, there has been just talks about just where to report stuff rather than how to talk to the individual about it.”*—Strength & Conditioning Coach (C)

“*I get a certain amount of funds per year to devote toward what we call professional development, so I usually utilize those funds for some of the continuing education credits and then we also get some funding to travel to conferences.”*—Sports Psychologist (E)

All three of the strength and conditioning coaches interviewed reported having either a certification or training in general nutritional counseling.

“*I do [nutritional] counseling on the side…work with a couple of people in the military who want to get fit or eat better…I'm not a registered dietitian. I don't have a nutrition certification…So I just work with general people or athletes…trying to optimize their nutrition.”*—Strength & Conditioning Coach (D)

Being educated and educating are different things, so participants were asked how confident they felt when relaying information to their athletes. Again, except for those who specialized in either field, all other staff reported feeling unprepared for dealing with an athlete alone, without making a report or calling in other staff for support.

“*I don't feel, myself, a hundred percent confident how to move on from [conversations] without having to reach out to someone. I think our main role…[is] more of an assess and refer [position]”*—Athletic Trainer (C)

“*If I was asked to do a standalone presentation on [nutrition], I would have zero confidence…I could jump in there, I could understand it…but then once I presented it, poof, it would go away.”*—Sports Psychologist (D)

“*When athletes come to me and tell me about what's going on…I'm happy to listen, but I don't know what to do, so it's kind of frustrating from that aspect.”*—Strength & Conditioning Coach (D)

“*From a nutrition standpoint, I am extremely confident in discussing to what my personal experience is.”*—Strength & Conditioning Coach (F)

### Integration

One of the biggest distinctions between these newly developed programs was whether the athletic department staff were integrated, or had regular, in-depth communication with each other on a regular basis. Some departments reported experiencing full staff collaboration, with regular meetings that involve all staff in the healthcare of individual athletes.

“*With every sport we have what's called a CARE meeting. So we basically review every single athlete and their health status every month. And in those meetings we have all of the health providers, so athletic trainers, strength coach, sports psych[ology], sports nutrition, sports med[icine], the coach.”*—Sports Dietitian (A)

“*We have staff meetings every week. We would have our athletic trainers. We would have somebody from the counseling center and we would have the dietician all there. So that we would sort of have rounds, like if you would say like medical rounds on our student athletes.”*—Sports Physician (C)

“*I think one of the reasons I came back to [School D] and one of the reasons it was attracted to me is because I do feel like there is a value here in interdisciplinary practice and recognizing that there needs to be collaboration to best meet the needs of the students.”—*Sports Psychologist (D)

Other schools reported little to no integration between coaching staff, nutrition staff, psychology staff and sports medicine.

“*The popular term in business and administration is now silos…you have silos in strength and conditioning. You have a silo of nutrition, a silo of sports medicine and they don't communicate well; It's like lipstick on a pig, we're just giving up this fancy title and we put up these cool looking graphics that show that we're communicating, but we don't.”*—Strength & Conditioning Coach (D)

“*I'll kind of work more closely with our dietitian. But I mean that we'll just be in communication with each other in terms of ‘here's what I'm doing.”'*—Sports Psychologist (D)

Some participants expressed their belief in the importance of keeping some separation between departments and referring out for extra help when needed.

“*So to me it's really important that as a professional we stay in our own lanes…Especially since we have the added resources.”* —Sports Psychologist (B)

### Engagement

Oftentimes the recurring resource and educational shortages at each university came down to the value that school administration, athletes and coaches placed on nutrition and mental health services within their department. Some reported mixed buy-in from coaches across athletics, which often dictated how often athletes were exposed to those services.

“*I think…our referral process needs to be overhauled. I think a lot of coaches just aren't really used to having nutrition and behavioral health on campus, so they don't really know when to refer athletes to us.”*—Sports Dietitian (B)

“*[Some coaches] meet every single week, and they go through it with the academic advisor, with sports medicine. Sometimes the dietitian is there…they go through each individual [student-athlete]…and the communication is there…it's sport to sport.”* —Strength & Conditioning Coach (D)

A few participants highlighted that a lack of education for the coaching staff in either nutrition or mental health may be one cause for these disparities.

“*If the school was more prepared or well-equipped on the nutrition side of things and could educate the staff…I think that would benefit everyone else…and I think that just comes from the university listening to what [the] athletics department has to say.”*—Strength & Conditioning Coach (C)

“*When athletes come to me and tell me about what's going on…I'm happy to listen, but I don't know what to do, so it's kind of frustrating from that aspect.”*—Strength & Conditioning Coach (D)

Stigma is still rampant in college sports, but many reported that more and more athletes were starting to vocalize their need for mental health attention.

“*Every year I feel like there are more people, more student athletes, more athletics staff that come out and are trying to get help for whatever they may be dealing with”*—Sports Physician (C)

“*[Mental health] just seems to be much more in the spotlight, and kids seem to be more open to sharing. Maybe because it's more prevalent.”*—Strength & Conditioning Coach (F)

### Perspectives

Any additional thoughts that did not fall into one of the above categories could be placed under “perspectives.” Many participants unveiled the approach that they or their institution is likely to take when caring for student-athletes. Some schools had a clear “performance first” mindset, while others emphasized looking at the whole student-athlete.

“*But at the end of the day, our main goal is to improve their performance. Any sort of disordered eating or mental health issues might be the barrier to that.”*—Sports Dietitian (B)

“*I could care less how much someone is squatting or running faster…if they hate their life…I think it's a neglected area in performance.”*—Strength & Conditioning Coach (D)

The remaining perspectives tended to focus around the meaning and importance of mental health, especially as it pertains to student-athletes.

“*[Mental health] is… an ‘umbrella term,' but there's a lot of people sitting underneath that umbrella.”*—Athletic Trainer (B)

“*I see mental health…on a continuum…on one end of the continuum you have what I would describe as well-being, and so that is when somebody is in optimal mental health…the other end…I would describe as ill-being.”*—Sports Psychologist (D)

“*[Mental health] is a major concern, and something that my staff and I try to address just from a, ‘Hey, we should keep an eye out for what I would deem red lights and yellow lights.' I'm a red light, green light, yellow kind of thing. What do we deem as yellows, what do we deem as greens? And how do too many yellows equal a red?”*—Strength & Conditioning Coach (F)

There was an insistence on a need for better and more frequent screening for student-athlete nutrition and mental health.

“*Looking at these PHQ-9 [depression scores] over a period of the course of their college careers would be helpful…But we just haven't looked at that yet.”*—Sports Physician (C)

### Discussion

From these results it is evident that all participants representing five schools were in the early stages of implementing nutrition and mental health programming into their athletic programs. All participants highlighted the relationship between nutrition, mental health and performance and the importance for athletic programs to address these needs (NCAA, 2020; Parks et al., 2016; Abbey et al., 2017; Rudick and Dannels, 2018). Nearly all staff members expressed a greater need for resources and staffing, and most reported wanting more educational materials for staff on nutrition and mental health information. Most participants also reported that, in general, there is a greater perceived interest in seeking these services by athletes, especially for mental health issues. This is consistent with a recent study of collegiate student-athletes (Way et al., 2021), in addition to screening, interventions to build mental health literacy are needed. One such intervention by Vella et al. (2021) demonstrates the possibility to enhance mental health literacy in athletes to build resilience. To better care for the entire athlete, it is important that athletic institutions continue to build upon these foundations (NCAA, 2020).

It is important to note that the schools with the resources to hire full-time sports psychologists and dietitians were more likely to have regular athlete-professional contact (e.g. weekly meetings, team talks, team events). This study and others found that schools who lack these staff rely on athletic trainers to recognize, report and follow-through on protocols for student-athletes' mental health issues, or to contact local dietitians for athletes with eating disorders (Baugh et al., 2020). In medical contexts, the ratio of staff to patients is associated with health outcomes, and the first empirical study of this in collegiate athletic programs found links between disparities in athletes' access to clinicians based on their NCAA division (Baugh et al., 2020). Compared with schools who rely on athletic trainers, those with full-time nutrition and mental health professionals likely have a more preventative approach to athlete care. Further research is needed to determine the extent to which staffing affects the health status of student-athletes (Baugh et al., 2020).

While there are promising developments being made in areas of nutrition (Reguant-Closa et al., 2020) and mental health (Tomalski et al., 2019; NCAA, 2020), there is still a need to engage athletes and reduce stigma without unloading extra responsibility onto both staff and athletes (Kroshus, 2016; Sontang-Padilla et al., 2018; Purcell et al., 2019; Baugh et al., 2020). In addition, COVID-19 has further limited the opportunities for schools to hire more staff or obtain greater resources (DePietro, 2020; Moody, 2020). Therefore, we have compiled a list of suggestions for improving nutrition and mental health within Division I athletic departments that require little to no extra cost or staff involvement.

### Recommendations

#### Provide a General Nutrition Plan for Athletes

Even if a school does not have a full-time dietitian on-hand, short nutrition guides can be made for athletes to plan meals for either on-campus dining or at-home cooking. Existing plans include the basic elements of a “performance plate,” as outlined by the United States Olympic Committee of Sports Dietitians and the University of Colorado (UCCS) (Reguant-Closa et al., 2020). It would also be helpful to list examples of options for each food group that can be found in the university dining halls or local grocery store(s). Research shows that competitive athletes are susceptible to diet fads and misinformation (Condon et al., 2007). Granting athletes access to a simple online resource that is universally accepted by the athletic department can give athletes a clear guideline to follow and may limit some unhealthy eating patterns. If possible, having a staff member give a talk to student-athletes about nutrition at the start of the academic year or before the start of a season may be helpful. For example, a school without a sports dietitian may be able to have a nutrition professor speak to athletes or ask an exercise science professor to speak on appropriate fueling and meal timing. Previous research has demonstrated that education interventions can improve nutritional knowledge and dietary behaviors (Abood et al., 2004; Valliant et al., 2012; Trakman et al., 2016).

#### Offer and/or Require Regular Mental Health Screening for Student-Athletes

A list of best practices by the NCAA strongly recommends written mental health screening tools to be administered prior to an athlete's participation in collegiate athletics (NCAA, 2020). Less than 40% of institutions reported having a plan to screen athletes for mental health disorders in 2016, and less than half actually administered written or verbal mental health screening tools to their athletes (Kroshus, 2016). Sudano and Miles (2016) similarly found that few schools utilize mental health screening tools. Given that athletes are less likely to seek out help (Moore, 2017) and are more susceptible to mental illness (Roberts et al., 2016), it is imperative that schools are regularly monitoring their players' mental health. The aforementioned NCAA website provides access to guides and scoring methods for several common mental health screening tools (NCAA, 2020). Brief mental health screening tools are available online and can be implemented into a brief virtual survey for athletes. For example, the General Anxiety Disorder Screener (GAD-7; Spitzer et al., 2006) or Patient Health Questionnaire (PHQ-9; Kroenke et al., 2001) measure symptom severity for anxiety or depressive mental health disorders in a series of questions. A survey platform, such as Google Forms (Google Drive, free to all “Education” users), Qualtrics ($1–5,000 per year, Seattle, WA), or Survey Monkey ($25–75 per user per month, San Mateo, CA) is needed in order to administer regular mental health surveys to athletes.

Although no comprehensive framework exists for supporting and responding to student-athlete mental health (Purcell et al., 2019), there have been published protocols that are meeting student-athlete needs (Tomalski et al., 2019; NCAA, 2020). An efficient mental health screening protocol for one university included administering a 5-min mental health survey after a 45-min mental literacy talk or activity session given by a sport psychology staff member (Tomalski et al., 2019). After surveying the athletes, follow-up meetings were conducted with any student-athletes who were flagged for potentially life-threatening situations (Tomalski et al., 2019). Meetings with less severe cases were scheduled in the weeks following (Tomalski et al., 2019). Optimally, it would be recommended that schools invent a similar protocol to carry out at the beginning of the academic year and re-administer the mental health survey(s) at least two to three times per academic year, preferably around times of higher stress such as midterm or final exams. One's mental health is likely to shift throughout the year(s), so it makes sense to continue monitoring athletes' mental states throughout their athletic career. A recent study found that those collegiate student-athletes who participated in some form of mental health screening were more satisfied with the availability of services and could help the practitioners offer services (Way et al., 2021).

#### Initiate a Student-Led Mental Health Space for Student-Athletes

Studies show that becoming part of a group can help individuals gain new perspectives and gain a sense of belonging, which may benefit mental health (Cintron et al., 1999). National organizations like Active Minds offer plans to help undergraduates run their own mental health groups and have been shown to improve mental health literacy and reduce stigma (Sontang-Padilla et al., 2018). Active Minds is a nonprofit organization that has chapters in over 550 college campuses across the United States with the goal of challenging the ways that mental health is normally discussed among adolescents and young adults (https://www.activeminds.org/about-us/mission-and-impact/). The nonprofit offers additional resources for student advocates, such as the Transform Your Campus Advocacy Guide or a guide for starting an Active Minds chapter. If the school does not already have a chapter, these programs could be introduced by athletics faculty to interested student-athletes. Schools with Active Minds chapters should try to foster a relationship between the chapter and athletics to build a program that targets student-athlete mental health. Even a more casual, social space is still likely to produce benefit for the athletes involved. For instance, one of the participants interviewed for this study reported hosting for the last few years a regular, student-run group of female student-athletes to discuss mental health and female body image concerns. After being initiated by the sports psychologist and dietitian, the athletes took control and continued their engagement with the group. Schools without these staff members could have an athletic trainer, school counselor or Professor of Psychology supervise group discussions, and discussion groups could be made around other topics as they relate to mental health (e.g. athletic identity, team cohesion, burnout, etc.).

Another participant spoke of a “Mental Health Allies” program at their institution. Student-athlete volunteers are trained by the department's sports psychologist in basic mental health literacy (e.g. symptoms for common mental health disorders like anxiety or depression, campus mental health resources) so that they can educate and provide support to their peers around mental health topics. The goal of this program is to gradually improve mental health literacy among students on campus and to reduce stigma around help-seeking behavior. Again, schools without a sports psychologist or other mental health professional on-staff can have a Professor of Psychology, school counselor or an athletic trainer trained in basic mental health protocols provide training for student-athlete “allies.”

#### Create General Nutrition and Mental Health Infographics to Post in Athletes' Spaces

Dietitians, sports psychologists, or athletic trainers can be asked to create simple infographics on general nutrition and mental health information for athletes, including listed resources to access information or support systems. Infographics have become a popular method of condensing and communicating information visually (Dunlap and Lowenthal, 2016), and are shown to benefit recall in college professors and students (Wansink and Robbins, 2016). Helpful guides to creating infographics exist online (Muir and Munroe-Chandler, 2020), and websites like Canva (Sydney, AU) provide aesthetic templates for presenting visual information at around $119 per year. Locating informational materials in common athletic spaces, like locker rooms, weight rooms and athletic buildings may increase the likelihood that athletes will notice and become familiar with the material. Although changes in behavior are not guaranteed, infographics are one way to increase awareness and comprehension of essential nutrition and mental health materials (Muir and Munroe-Chandler, 2020).

### Limitations

Colleges and universities have had to make large adjustments to the 2020–2021 COVID-19 pandemic, including altering learning procedures, codes of conduct, and letting go of excess staff (DePietro, 2020; Moody, 2020). Meanwhile, most schools have had to offer additional financial support to students whose families were hit hard by the pandemic (Moody, 2020). Student enrollment in colleges has been down since the start of the pandemic, leaving some schools with no choice but to shut their doors, or to lay off and cut benefits to large amounts of staff (DePietro, 2020). When it comes to athletics, funds are expected to be limited for non-revenue sports, and some programs or conferences are not expected to see competition for the foreseeable future (Moody, 2020). Some participants reported greater amounts of stress since the start of the pandemic, in large part due to limited resources, increased responsibility for fewer staff, and an unknown path for return to normal athletic competition. Interviews for this study were conducted between June and September of 2020, in the midst of the pandemic where schools were still sorting out their plans for fall semester. Therefore, it should be considered that the reported dynamics within each NCAA Division I athletic department may have been different than that of a “normal” semester. However, conversations with some staff seemed to echo that the status of the nutrition and mental health programming within the departments either remained stagnant or continued to grow during the COVID-19 pandemic, despite the new constraints.

Another limitation for this study was that not all of the eligible staff members from each school's Athletic Staff Directory were sent an interest email for the interview. In particular, Head and Assistant Coaches for different sports were purposely selected to allow for diversity in sports representation (e.g. high-monetization versus low-monetization sports). It is possible that the study population would have been larger and/or more diverse if all eligible staff from each institution were contacted. In addition, the numbers of each type of staff member were not consistent across schools, mainly due to a lack of responses to the initial interest email. However, this was not seen as a major issue since saturation of responses still occurred among the seventeen interviewed participants.

## Data Availability Statement

The raw data supporting the conclusions of this article will be made available by the authors, without undue reservation.

## Ethics Statement

The studies involving human participants were reviewed and approved by Elon University Institutional Review Board. The patients/participants provided their written informed consent to participate in this study.

## Author Contributions

EM, CK, and EH conceived of the presented idea. EM carried out the project (recruitment, interviews, and data analysis) with support from CK and EH. All authors discussed the results and EM wrote the final manuscript with support from CK and EH. All authors contributed to the article and approved the submitted version.

## Conflict of Interest

The authors declare that the research was conducted in the absence of any commercial or financial relationships that could be construed as a potential conflict of interest.
